# The Constructive Neutral Evolution of Behaviour

**DOI:** 10.1002/ece3.71736

**Published:** 2025-07-10

**Authors:** Andrew M. Catherall‐Ostler, Tanmay Dixit

**Affiliations:** ^1^ Homerton College University of Cambridge Cambridge UK; ^2^ Department of Zoology University of Cambridge Cambridge UK; ^3^ FitzPatrick Institute of African Ornithology, Department of Biological Sciences University of Cape Town Cape Town South Africa

**Keywords:** behaviour, constructive neutral evolution, molecular evolution, mutations, nonadaptive evolution, trait evolution

## Abstract

There is an unrecognised disagreement between those studying molecular evolution and those studying behavioural evolution. Only natural selection is ordinarily thought to systematically increase behavioural complexity, but molecular biologists are increasingly recognising a second driver of complexity—constructive neutral evolution (CNE). CNE occurs when a system contains components that buffer the effects of otherwise‐deleterious mutations, allowing such mutations to spread. This increases the complexity of the system due to the dependency that now exists between the buffering component and the mutated component. A probabilistic ‘ratchet’ means this process is more likely to repeat itself than reverse, and hence complexity increases without necessarily any gain in function. Here, we argue that CNE operates not only at the molecular level, but also affects whole organism behaviour. We suggest that behaviour's polygenic architecture, flexibility, and ability to mitigate the impact of otherwise‐deleterious mutations mean that behaviour is particularly likely to evolve via CNE. We summarise the evidence that supports this hypothesis as well as suggesting how it could be tested further. We conclude that CNE must be considered alongside selective explanations for increases in behavioural complexity.

## Graphical abstract

We explain how constructive neutral evolution (CNE) can lead to the evolution of nonfunctional behavioural complexity, highlighting a mechanism that is unknown to behavioural ecologists despite being frequently considered by molecular biologists. We summarise indirect evidence that suggests behavioural CNE has occurred and outline how the hypothesis can be tested more directly.

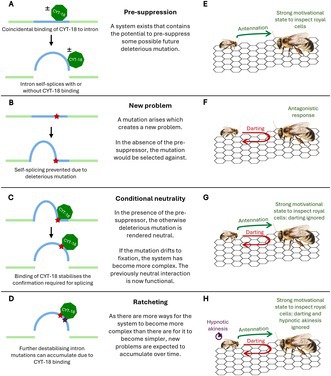

## 1 Introduction

Living things are interesting because of their complexity, and natural selection is usually recognised as the only force capable of systematically increasing biological complexity (Brandon 1990; Charlesworth et al. 2017; Futuyma 2017; Williams 1966). Recent empirical investigations have documented in detail the conditions under which natural selection causes phenotypic complexity to increase, such as coevolutionary arms races or intersexual selection (Adami 2002; Choi et al. 2022; Dixit et al. 2022, 2023). Animal behaviour can be particularly complex, and ‘adaptationist’ approaches in behavioural ecology have successfully revealed ultimate causes for many complex behaviours (Cuthill 2005; Davies et al. 2012). However, for molecular and cellular phenotypes, an alternative, nonadaptive route to complexification has long been recognised: constructive neutral evolution (CNE; Muñoz‐Gómez et al. 2021; Stoltzfus 1999, 2012; Torri et al. 2022; Wideman et al. 2019). Here, we argue that if CNE can increase the complexity of subcellular phenotypes, it can also increase the complexity of whole organism behaviour.

## 2 Section 1: The Process of Constructive Neutral Evolution

### 2.1 What Is Constructive Neutral Evolution?

Constructive neutral evolution (Stoltzfus 1999) is a process by which a system's complexity increases without the need for any positive selection: the system becomes more complex without any increase in function. ‘Complexity’ lacks a single well‐accepted definition (Adami 2002; Ladyman et al. 2013), but in the context of CNE we are interested in changes in the number of interactions required between a system's components for the system to function properly (the ‘combinability’ dimension of complexity *sensu* Rebout et al. (2021)). If the number of interactions required for a system to function increases, it has become more complex.

For our purposes, a biological system is any set of biological entities—including biomolecules, cells, tissues, organs or organisms—that interact to carry out a specific function. Biological systems often consist of many components, and an even greater number of interactions between these components. The human proteome, for example, contains around 20,000 proteins (Omenn et al. 2024): approximately 1.3 million pairwise interactions between them have already been identified, with many more still to be discovered (Wilkins et al. 2024). Many interactions serve useful cellular functions, such as the binding of a hormone to its receptor. Other interactions, however, are ‘neutral’—that is, their presence or absence leads to no change in fitness. For example, the human proteome involves many interactions thought to be neutral and caused by chance structural complementarity (Bergeron‐Sandoval et al. 2016; Krishnan et al. 2022; Landry et al. 2013; Levy et al. 2012), with recent estimates suggesting these may comprise as many as 20% of all protein–protein interactions (Ghadie and Xia 2019, 2022; for a detailed example see Box 2).

**FIGURE 1:**
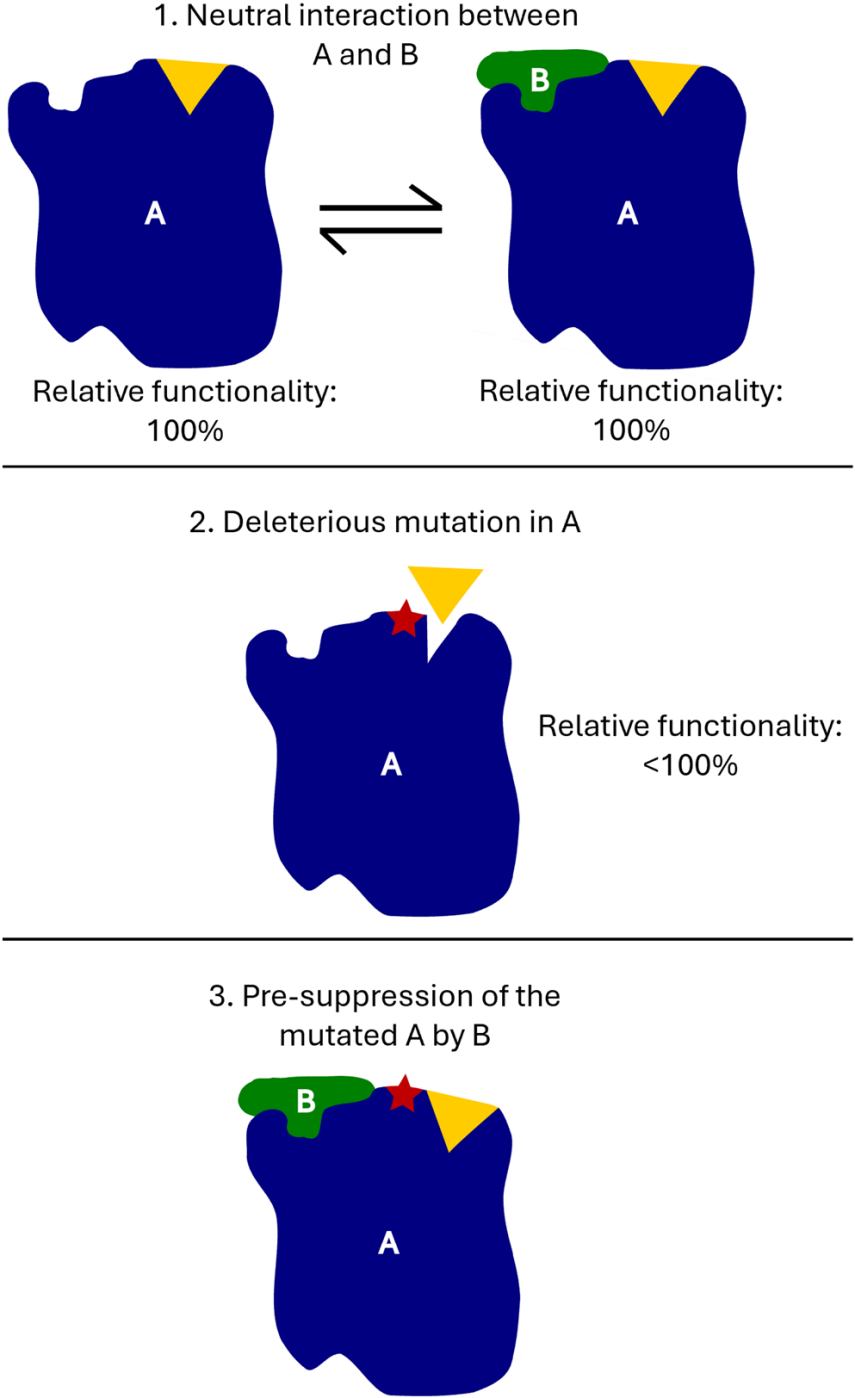
Process of constructive neutral evolution. In Step 1, protein A and its substrate (yellow triangle) are complementary; there is also a coincidental binding between protein A and protein B. In Step 2, a mutation occurs that prevents A from binding to its substrate. In Step 3, we see that the coincidental binding of B to A has rendered this mutation conditionally neutral, as the resulting conformational change allows substrate binding to still occur. The system has become more complex as A is now dependent on B.

Importantly, some of these neutral interactions may, by chance, be able to rescue an organism from the consequences of an otherwise‐deleterious mutation (Stoltzfus 1999). CNE (Figure 1) occurs as follows:

1. Consider two proteins, A and B, which currently interact neutrally—that is, they bind to each other, but this coincidental binding has no functional benefit.
2. A deleterious mutation occurs that changes the shape of protein A such that it can no longer perform its normal function. Ordinarily, this mutation would be removed by selection.
3. However, if the coincidental binding of B to A happens to stabilise the shape of the mutated A, then the otherwise‐deleterious mutation is rendered neutral.

Through genetic drift, the mutation in A could either be lost or spread to fixation. If it does spread to fixation, then the system (here, the proteome) has become more complex, as the number of interactions required for the system to function properly has increased. The interaction between A and B is now functional and will be preserved by purifying selection; the system has increased in complexity but not in functionality.

CNE is not itself an evolutionary force equivalent to selection or drift (Box 1). Instead, it is a phenomenon that occurs when three conditions are met. First, a neutral interaction must exist that prevents the deleterious effects of some future mutations—this is known as ‘pre‐suppression’. The presuppressor—the component that does the presuppressing, such as protein B in the example above—is not itself necessarily neutral. Protein B will presumably perform a useful role in the cell. What is ‘neutral’, prior to the mutation in A, is the unselected, coincidental binding of protein B to protein A. Such coincidental binding is likely very common (Ghadie and Xia 2019, 2022): molecules that do not interact functionally often show high levels of coincidental structural complementarity (Launay et al. 2017), and high concentrations inside the cytoplasm (Majumder et al. 2016) mean even low‐affinity interactions occur frequently (Acerenza and Graña 2006; Wirth and Gruebele 2013).

BOX 1 **The place of CNE in evolutionary theory.**

Have CNE advocates really discovered a new evolutionary phenomenon or is CNE just a complicated re‐description of the already well‐known fact that neutral mutations can drift through populations (Speijer 2011)? We have found that the theoretical status of CNE is a potential area of confusion and that it can be clarified by distinguishing between *forces* and *phenomena* in evolution.

Evolutionary change is caused by four fundamental forces, each of which affects allele frequencies in different ways: mutation, migration, selection and drift (Fisher 1930; Haldane 1932; Stephens 2004). CNE is not such a force. Equally, CNE is not a property of a system or trait, such as plasticity (in which variation in a trait, such as a behaviour, is influenced by environmental variation), or an indirect genetic effect (in which trait variation in focal individuals is influenced by genetic variation in other individuals). Instead, CNE is a phenomenon that arises when three of the four fundamental evolutionary forces—mutation, drift and (purifying) selection—act on a system that has particular conditions, namely presuppression and a bias towards the generation and/or toleration of new variants that contribute additional complexity rather than those that reduce complexity. Properties of systems, such as behavioural plasticity or indirect genetic effects influencing organismal traits, may contribute to CNE through presuppression (as discussed later in this article). CNE therefore has the same theoretical status in evolutionary biology as other named phenomena in which particular evolutionary forces acting on systems that meet particular conditions produce predictable results, such as runaway selection, Müller's ratchet, genetic hitchhiking or plasticity‐first evolution.

CNE is not the only way in which complexity could increase despite a lack of positive selection. Mutation and drift alone, in the absence of the presuppressors or probabilistic ratchets discussed below, may by chance increase complexity. However, the key difference is that systems that meet the criteria for CNE to occur are expected to systematically increase in complexity, whereas drift alone, being a process of random sampling, cannot in and of itself cause systematic increases. Such increases in complexity must be generated by nonrandom processes, such as directional selection (Adami 2002; Choi et al. 2022; Dixit et al. 2022, 2023) or CNE.

The CNE hypothesis should also not be confused with claims that there is a law‐like tendency towards complexity increases in evolution (McShea et al. 2019; McShea and Brandon 2010). CNE‐driven increases in complexity will only occur in systems where the above conditions are met. Indeed, if a system contained a bias towards the generation and/or toleration of new variants that *decrease* complexity, then an equivalent process of reductive neutral evolution (RNE) could be expected to reduce complexity over time (Stoltzfus 2015).

Second, CNE requires ‘conditionally neutral’ mutations to occur. These are mutations that are deleterious in one genetic background (due, in CNE, to the absence of a presuppressor), but not deleterious in other genetic backgrounds (due, in CNE, to the presence of a presuppressor). Mutations that are conditionally neutral due to such epistasis are likely very common (Raman et al. 2016; also see Box 2), with an estimated 90% of amino acid substitutions being conditionally neutral in protein evolution (Breen et al. 2012). There are also many cases where the *adaptive* evolution of a protein (c.f. the nonadaptive evolution in CNE) relied on the prior chance fixation of a neutral mutation (e.g., Gong et al. 2013; Lunzer et al. 2010; Ortlund et al. 2007; Tóth‐Petróczy and Tawfik 2013).

BOX 2 **Evidence of CNE in molecular biology.**

CNE has been proposed to explain the evolution of many biochemical and cellular phenomena (Table B1).

**TABLE B1:** Cellular and subcellular traits for which CNE models for their origin and/or diversity have been proposed. These include both common properties of cellular and genomic organisation and unusual features of particular taxonomic groups.

Trait	References
Traits common to many organisms	
Retention of duplicate genes	Després et al. (2024), Lynch and Force (2000), Stoltzfus (1999)
Chaperone proteins	Schulz et al. (2022)
Complexity of the ribosome and spliceosome	Doolittle et al. (2011), Gray et al. (2010), Lukeš et al. (2011), Stoltzfus (1999)
Protein–protein interaction interfaces and protein complexes	Després et al. (2024), Prokopchuk et al. (2023), Schulz et al. (2022)
Endosymbioses and horizontal gene transfer	Jones et al. (2022), Sitaraman (2020)
Long non‐coding RNAs	Palazzo and Koonin (2020), Palazzo and Lee (2018)
RNA interference	Torri et al. (2022)
Junk DNA	Fagundes et al. (2022)
Diversification in RNAse P	Weber et al. (2014)
Extensions of mitoribosomal proteins	Shikha et al. (2025)
A ➔ I mRNA editing	Xie and Duan (2025)
Traits found in a restricted group of organisms	
Ciliate gene scrambling and multiple nuclei	Boscaro and Keeling (2023), Lukeš et al. (2010), Smith and Keeling (2016), Stoltzfus (1999)
Trans‐splicing, polycistronic transcripts and RNA editing in kinetoplastids and dinoflagellates	Covello and Gray (1993), Flegontov et al. (2011), Gray et al. (2010), Lukeš et al. (2009, 2011), Moreira et al. (2015), Smith and Keeling (2016)
Mammalian cellular clock	Sancar (2008)
Cellulose synthesis hetero‐oligomeric complexes in mosses and seed plants	Li et al. (2019)
Melanocortin receptor gene family	Dores (2013)
Dicistronic transcription unit in *Tribolim castaneum*	Song et al. (2020)
Chloroplast RNA metabolism in *Chlamydomonas*	Lefebvre‐Legendre et al. (2014)
Obligate halophilicity in proteobacteria	Deole et al. (2013)
Role of *MECP2* in neurogenesis	Bird (2020)

**FIGURE 2:**
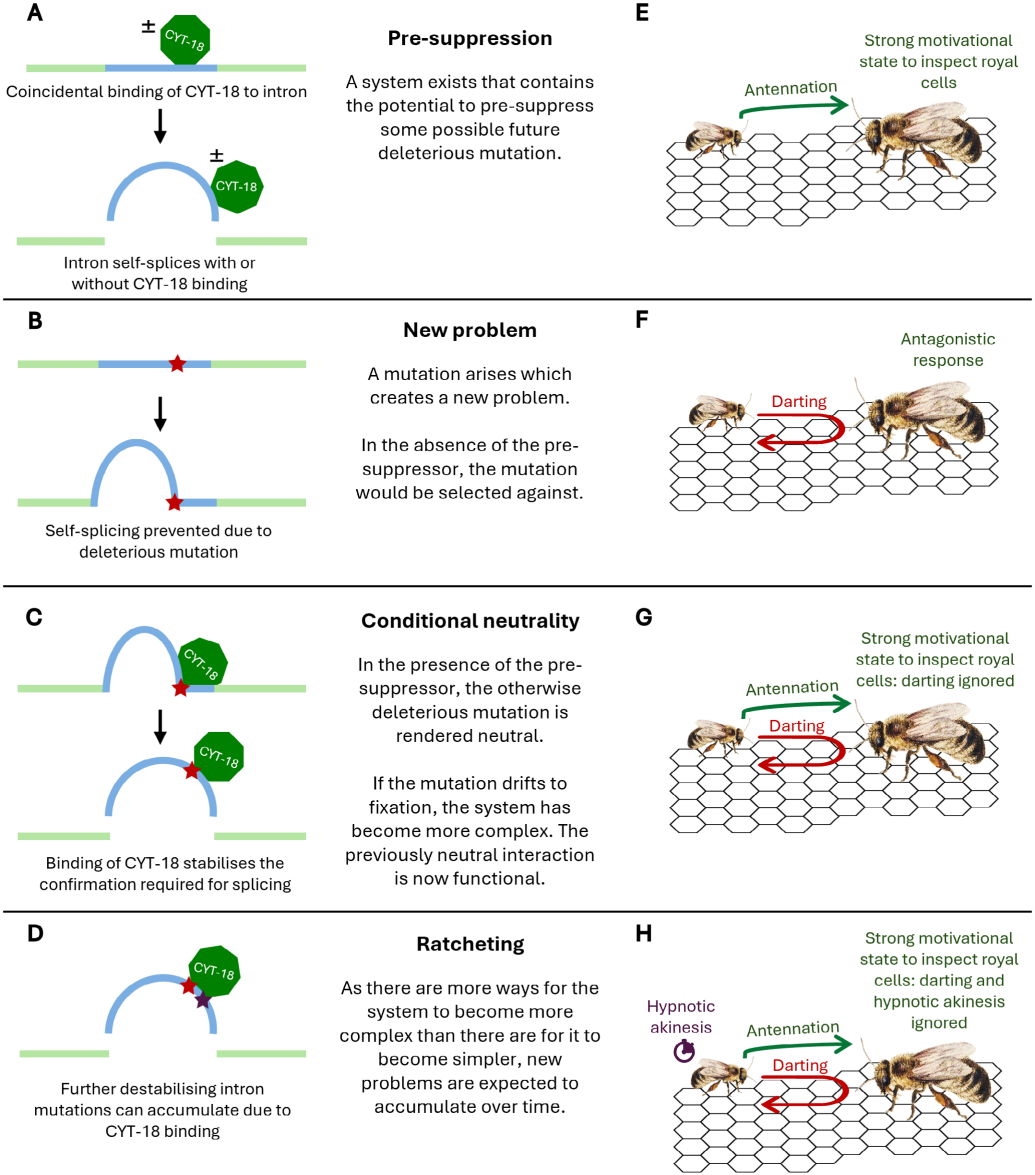
Left (A‐D): A CNE model for the evolution of introns in the mitochondrial genome of *Neurospora* (see Box 2 for details). Right (E‐H): A CNE model for the evolution of POP behaviours in stingless bees (see main text for details). Presuppressors (CYT‐18 and its intron‐stabilising capacity; the strong motivational state induced by antennation) are colour‐coded dark green, initial conditionally neutral mutations are coloured red, and subsequent conditionally neutral mutations that are also presuppressed by the presuppressor (and thus ‘ratchet up’ complexity) are coloured purple.

For example, consider the evolution of *Neurospora*'s mitochondrial genome (Figure 2A–D). This contains some introns that self‐splice (the ancestral state) and others whose excision depends on the binding of the protein CYT‐18 (Lambowitz and Perlman 1990). A conventional ‘problem‐then‐solution’ explanation (e.g., Paukstelis and Lambowitz 2008) for the origin of this complicated system is that a mutation arose in an intron which prevented it from self‐splicing. This mutation was deleterious, but somehow this new ‘problem’ spread to fixation. Later another mutation arose in *CYT‐18*, allowing CYT‐18 to assist excision of the defective intron: this ‘solution’ was favoured by selection and rapidly fixed (Paukstelis and Lambowitz 2008). CNE advocates argue that this conventional problem‐then‐solution model is implausible (Gray et al. 2010). If defective introns are deleterious, they are likely to be lost rather than spread to fixation. Even if they do spread to fixation, then the population must somehow be able to survive long enough for a genetic solution to arise (Gray et al. 2010).

A CNE mechanism, by contrast, would involve the solution arising before the problem (Gray et al. 2010). Stoltzfus (1999) proposed the following scenario. In some species, CYT‐18 does not contribute to intron splicing but still happens to bind to conserved intron structures (Stoltzfus 1999; Paukstelis and Lambowitz 2008). This implies that the ability of CYT‐18 to bind to and assist defective introns may have been present *before* the defective introns themselves (Figure 2A). Such presuppression would mean that when the mutation that prevented self‐splicing occurred (Figure 2B), CYT‐18 instantly masked the consequences of this otherwise deleterious mutation, allowing it to drift to fixation (Figure 2C). The excision of this intron was now dependent on CYT‐18, increasing the complexity of the system. As it is more likely that random mutation would cause further degeneration of this (or other) introns rather than restoring self‐splicing, introns would become more and more dependent on CYT‐18 over time—that is, successful splicing in the absence of CYT‐18 binding becomes less and less likely (Figure 2D). The presuppression provided by CYT‐18 would not just be the solution to the problem of intron degeneration but also its ultimate cause. This solution‐then‐problem hypothesis avoids requiring populations to somehow survive in fitness valleys until solutions arise and provides a potential explanation for how pointless complexity could accumulate over time.

CNE may occur through Steps 1–3 and then go no further, as proposed for the evolution of marsupial mt‐tRNA editing in which only a single base pair is edited (Börner and Pääbo 1996). Equally, it is possible that back‐mutation may revert the system to its original simpler state. CNE advocates argue, however, that there are good reasons to think this process is more likely to repeat than reverse, a tendency called ‘ratcheting’.

### 2.2 Ratcheting

CNE is termed ‘constructive’ as it can repeatedly increase, ratchet‐like, a system's complexity over time (Lukeš et al. 2011; Stoltzfus 1999, 2012). The ‘ratchet’ is probabilistic, arising from a bias in the distribution of future mutational effects: there are more ways for the system to become more complex than there are for it to revert to its original, simpler state (Gray et al. 2010; Lukeš et al. 2011). The only way for the system to return to its original lower level of complexity would be for the conditionally neutral mutation (i.e., the mutation that destabilised protein A in the example above) to revert. However, there are many more ways that the process outlined in Steps 1–3 above could repeat either at this locus or elsewhere in the genome (Lukeš et al. 2011; Stoltzfus 2012). When the number of potential mutations that could increase complexity are greater than the number that could reduce complexity, complexity will be expected to increase (Doolittle et al. 2011; Gould 1996). In addition to this probabilistic argument, some new dependencies that arise may themselves presuppress further mutation. For example, the origin of an mRNA editing system via CNE (Box 2) would enable the fixation of further mutations that require editing. Finally, systems generally tolerate the addition of components better than their removal (Glazenburg and Laan 2023; Saunders and Ho 1976; Soyer and Bonhoeffer 2006), furthering the bias towards increasing complexity.

### 2.3 CNE Beyond the Molecular Level

Almost all prior applications of CNE have been at the molecular or cellular level (Box 2). Though not without controversy (e.g., Speijer 2010, 2011; responded to by Doolittle et al. 2011), CNE is increasingly accepted as a potential explanation, alternate to or combined with selection, for the origins and/or elaboration of molecular complexity (e.g., Dodbele et al. 2018; van Esveld and Huynen 2018; Sloan and Wu 2016; Smith 2016) and has recently been demonstrated experimentally (Després et al. 2024). If CNE can affect cellular phenotypes, there seems no reason why it could not affect phenotypes at higher levels of biological organisation, provided presuppression, conditionally neutral mutation, and ratcheting occur. Brunet and Doolittle (2018), Brunet (2022), and Parker (2024) provide several examples of how physiological complexity in multicellular organisms might have evolved via CNE. In this paper, we explain how whole organism behaviour could also evolve via CNE, and why behavioural traits might be particularly prone to CNE‐driven complexification.

## 3 Section 2: How Behaviour Could Evolve via CNE

### 3.1 Is Behaviour Ever Pointlessly Complex?

CNE explains why some cellular machinery and processes appear needlessly complex. For instance, in *Trypanosoma brucei*, primary mRNAs do not encode functional proteins. Successful gene expression instead requires a complex posttranscriptional editing system involving over 1000 guide RNAs and 70 proteins to convert these nonfunctional transcripts (‘cryptogenes’) into mature mRNAs that code for functional proteins. Over 3000 particular uridine residues are inserted and deleted across the transcriptome—but all of this complexity exists merely to restore transcripts to their ancestral, premutated functional state (Aphasizheva and Aphasizhev 2016; Lukeš et al. 2011; Stoltzfus 1999). This ‘apparently superfluous detour’ (Malek et al. 1996) is not unique to trypanosomes: many other cellular processes (Table B1) have been likened to Rube Goldberg machines, named after the cartoonist who amused his readers by designing very complicated machines to carry out simple tasks.

Animal behaviour also sometimes appears unnecessarily complex. For example, consider the provisioning and oviposition process (POP) in stingless bees. In *Melipona quadrifasciata anthidioides* , workers constructing royal cells bow down before the inspecting queen, allowing her to tap them on the vertex. Workers then tremble repeatedly, inserting their forebody into the cell while the queen taps them firmly with her antennae and forelegs. Alternately dashing in and out, the workers discharge larval food into the cell, before one lays a trophic egg. Eventually, the queen enters and eats the egg and some of the food they have provisioned. The queen then lays her own egg and departs; some workers remain behind to seal the cell with a cap (Sakagami et al. 1965).

This is one of the simpler versions of stingless bees' POP rituals. In the genus *Plebia* there are approximately 30 species, which exhibit considerable interspecific and intraspecific diversity in POP behaviour. In some species the queen and workers grasp each other with their forelegs, in others they engage in mutual pushing, in some they do both, and in others they do neither (Drumond et al. 2000). In some, but not all, species the workers ‘dart’—they rush towards the queen before immediately retreating (Drumond et al. 2000; Sommeijer and Bruijn 1984). In many species, the ritual includes a period of ‘hypnotic akinesis’: workers lower their head before the queen and, as if hypnotised, turn their head slowly from side to side before freezing still.

The POP ritual likely helps ensure queen reproduction is coordinated with worker activities (Sommeijer and Bruijn 1984) and/or allows the queen to exert dominance over workers (Zucchi 1993), but the complexity of the ritual seems gratuitous (Grüter 2020; Zucchi 1999). The variation in POP rituals between species suggests that each component behaviour is not essential, as does the fact that queens placed in colonies of heterospecific workers often still manage to successfully oviposit (da Silva 1977; Sakagami 1982). It seems, therefore, that these rituals are perhaps a whole organism analogue of the overly complex RNA and intron editing ‘rituals’ described above (Figure 1; Box 2) and serve as an example of how unnecessary complexity might manifest itself in a behavioural context.

### 3.2 How Could Behavioural CNE Work?

In this section, we describe how CNE could drive the evolution of behavioural complexity. Of course, CNE could affect molecular interactions between gene products as described in Section 1, and if these gene products are involved in regulating behaviours, then behaviour would evolve via CNE (for a proposed example, see Bird 2020). As this is conceptually identical to previous models of CNE, we do not discuss this possibility further.

However, the components in a CNE model need not be proteins or other gene products. Interactions between any of the phenotypic components involved in the production of an individual's behaviour could lead to CNE: interactions between and among neurons, endocrine glands and effector organs could all presuppress otherwise deleterious changes. Beyond the individual level, we also now recognise that indirect genetic effects are a key component of the genetic architecture of behaviour (Hunt et al. 2019; Santostefano et al. 2017): genes in one individual frequently affect the phenotype of other individuals. Thus, a general scenario of behavioural CNE could occur as follows:

1. A behavioural system exists in which there are interactions between its ‘components’. The ‘components’ may be physiological structures or processes within an individual, or different individuals interacting socially.
2. Some of these interactions happen to prevent the consequences of otherwise deleterious mutations (presuppression).
3. When such a deleterious mutation occurs, the presuppressive interaction suddenly becomes useful as it neutralises the fitness costs of the novel mutation. This mutation may drift to fixation, leading to the system becoming more complex: it has gained an additional dependency (two components now *must* interact for the organism to be as fit as it was previously, otherwise the effects of the deleterious mutation will be realised).
4. This process is more likely to repeat itself (ratcheting) rather than revert the system to its previous state. Hence, behavioural complexity increases despite a lack of any necessary increase in function.

### 3.3 Social Behaviour—A Possible Case Study of CNE

Social behaviour is often remarkably complex (Strassmann and Queller 2007). Many adaptive explanations for the complexity of social behaviours have been proposed (reviewed in Freeberg et al. 2012), and these explain the evolution of many examples of elevated behavioural complexity as a result of ecological or sexual selection. In some cases, however, CNE having generated *pointless* complexity may provide a more plausible explanation than natural selection having generated *functional* complexity.

As a case study to illustrate how CNE in behaviour might occur, we return to the provisioning and oviposition process (POP) in stingless bees (Figure 2E–G). This is a promising candidate for behavioural CNE because there is a huge diversity of complex POP rituals among stingless bees which seem to accomplish essentially the same task. Of course, such variation in behaviours could arise due to factors such as interspecific (Yoder and Nuismer 2010) or intraspecific coevolution (Dixit 2024); here we merely attempt to show that neutral, nonfunctional origins for such variation should also be considered.

A verbal model of POP evolution by CNE is as follows. Let us assume that the original function of POP was for workers to notify the queen once brood cells have been constructed (Sommeijer and Bruijn 1984). As the POP is a synapomorphy of stingless bees, with sister taxa exhibiting much simpler reproductive behaviours (Melo 2020; Noll 2002), ancestral POP ‘rituals’ were almost certainly simpler: perhaps workers just walked up to the queen at her resting place and touched her rapidly with their antennae (antennation; Figure 2E). This worker behaviour will have co‐evolved with queen behaviour: just as workers would have had a behavioural rule saying, ‘when the royal cells are ready, antennate the queen’, the queen would have needed a response rule saying, ‘when antennated by a worker, cease what you are doing and become strongly motivated to oviposit’. Given this simple antennation–recruitment system, imagine that a mutation arose that caused the darting behaviour present in some stingless bees—after antennating the queen, workers suddenly dash forward again to the queen before immediately retreating (Sommeijer and Bruijn 1984). Before the evolution of the antennation–recruitment system, this darting behaviour would likely have been deleterious, since darting is typically an antagonistic behaviour (Figure 2F; Sommeijer and Bruijn 1984; Tóth et al. 2003; Zucchi 1993) that could spark undesired physical conflict. However, the queen's response rule to the antennation would mean that darting happens at a time when she is highly motivated to ignore other stimuli and instead advance to the cells and oviposit. The otherwise deleterious mutation that leads to darting is conditionally neutral—provided that antennation occurs, the behaviour has no effect on fitness (Figure 2G). The mutation underpinning darting could then drift to fixation, leading to the POP system becoming more complex. While antennation and the queen's response rule initially evolved for another function (ensuring that oviposition occurs after brood cells are constructed), they are now also functional in suppressing the otherwise deleterious effect of darting. We note that, for our purposes, whether darting actually was the first additional component of the POP to evolve is immaterial; we use it merely to illustrate how conditional neutrality could allow behavioural systems to increase in complexity without an increase in function.

As with molecular CNE, there is only one way the system could revert to its lower level of complexity: a reversal of the original mutation that stimulated workers to dart. There are, however, many ways for it to become more complex (Figure 2H). In some lineages, mutations could occur which make the worker contact the queen with its front legs during many frequent darts (e.g., *Nannotrigona testaceicornis* ); in others mutations that predispose the mandibles to open (e.g., *Plebeia*, *Trigonisca pediculana* ) or open and close while antennae are shook and heads rapidly raised and lowered (e.g., *Paratrigona*) (Zucchi 1993). Further odd worker behaviours, such as the previously mentioned hypnotic akinesis (Figure 2H), could accumulate (Appendix S1): so long as they are not highly costly (e.g., they are short and energetically inexpensive, and so have little effect on colony success and the workers' inclusive fitness) and co‐occur with antennation behaviour, then the queen's drive to respond to antennation will ensure that these other behaviours are not selected against. The logic underlying this argument is similar to Sakagami's (1982) original hypothesis for the origin of the POP: his model in which ‘odd’ behaviours evolve due to conflicting motivational drives within workers can be rephrased in terms of CNE (Appendix S1).

Overall, just as chaperone proteins can allow destabilising mutations in other genes to spread that otherwise would not in molecular CNE (Schulz et al. 2022), the motivational states and behaviours of workers and queens during POP behaviours could allow mutations that affect behaviour to spread that otherwise would not. In both cases, the systems become reliant on the presuppressors (chaperones in molecular CNE; antennation behaviour and its resultant motivational states in behavioural CNE). In both cases, the complexity of the system has increased due to an additional interaction that must now take place between the trait created by a conditionally neutral mutation (generating altered proteins structure in molecular CNE; darting in behavioural CNE) and the presuppressor. In both cases, further increases in complexity are possible if further conditionally neutral mutations spread. Complexity would increase whether measured by the number of features in the system (e.g., new degenerated regions of a proteins surface in molecular CNE, new worker behaviours such as darting or hypnotic akinesis in behavioural CNE) or by the number of interactions (due to the new interactions required between these new features and the presuppressor).

Examples of seemingly pointlessly complex social behaviours are not limited to reproductive rituals. Parental care behaviours vary considerably in complexity between taxa. In burying beetles (*Nicrophorus* spp.), there is interspecific variation in the dependence of offspring on care: experimentally preventing posthatching care causes brood failure in some species, while in others larval survival rates only decrease slightly, or even increase due to the absence of infanticidal brood reduction (Capodeanu‐Nägler et al. 2016; Prang et al. 2022). These differences are due to variation in the dependence of offspring on parental feeding—burying beetle parents regurgitate predigested carrion to their young (Capodeanu‐Nägler et al. 2016, 2018). Parental feeding is a synapomorphy of *Nicrophorus*, and hence burying beetles likely evolved from a species in which larvae were nutritionally independent from parents, with selection favouring parental care to protect offspring from intruders (Eggert and Müller 1997; Suzuki and Nagano 2006; Trumbo 1992). While ecological and life history factors explain some of the variation in parental care, an additional hypothesis is that the complexity of parental care increases by CNE:

1. Ancestrally, parents are selected to care for offspring—for example, remaining with the brood after oviposition.
2. While remaining with the brood, parents perform some behaviours which happen to presuppress a mutation in larvae which would otherwise be selected against. For example, when feeding on the carcass themselves, any residual oral fluid (containing digestive enzymes) transferred to the carcass may increase its digestibility for larvae. This is plausible given that extraoral digestion was likely present in the ancestor of *Nicrophorus* (Lawrence and Newton 1982).
3. A mutation in larvae that reduces their ability to bite tough carcass flesh (e.g., a reduction in jaw strength) may now be rendered effectively neutral (because the residual oral fluids compensate for weakened jaws) and drift to fixation. Offspring are now dependent on the previously incidental parental behaviour of transferring oral fluid to carcasses (Lawrence and Newton 1982). Oral fluid transfer is now a crucial part of the system due to its interaction with reduced larval jaw strength: this new dependency represents an increase in the system's complexity.
4. As the presuppressive effects of parental feeding behaviour on offspring feeding morphology and behaviour continue to shield otherwise‐deleterious mutations in offspring from selection, this process is likely to repeat itself and enhance the dependency of offspring traits on parental behaviours.

The origin of some parent–offspring interactions by nonadaptive processes explains otherwise puzzling levels of dependency: even when provided experimentally with easily ingestible liquefied carrion, *N. orbicollis* larvae generally do not survive without the transfer of parental oral fluids (Capodeanu‐Nägler et al. 2018). Digestion in *N. orbicollis* has seemingly become unnecessarily complex, especially given that carrion is an easy‐to‐digest resource (Tallamy and Wood 1986). Larvae of other *Nicrophorus* species—including likely the ancestor of *N. orbicollis* —can digest carrion independently, suggesting that incidental parent–offspring fluid exchange may have presuppressed the loss in *N. orbicollis* larvae of the required oral digestive enzyme or symbiont (Capodeanu‐Nägler et al. 2018).

If parental care acts as a presuppressor, then it should be associated with a decrease in purifying selection on otherwise‐deleterious mutations. Recent experimental work in burying beetles has supported this prediction, showing that parental care is indeed associated with increased genetic variation, likely because the shielding effect of parental care renders otherwise‐deleterious mutations neutral (Snell‐Rood et al. 2016). When parental care was removed, much genetic variation was swiftly lost (Mashoodh et al. 2023; Pascoal et al. 2023). This was not explained by demographic effects, and instead suggests that the system had become dependent on parental care, exactly as predicted by CNE.

### 3.4 Is Behavioural CNE Likely to Occur?

Gould and Lewontin (1979) suggest that merely demonstrating that a particular phenomenon could occur in evolution is unremarkable, as ‘in natural history, all possible things happen sometimes’. For CNE, however, demonstrating possibility is a significant contribution to behavioural ecology—since natural selection is generally considered the only possible explanation for systematic increases in behavioural complexity, just as was previously the case with molecular complexity (Stoltzfus 1999, 2012). Nevertheless, we now consider how common behavioural CNE is likely to be.

Arguments can be made for CNE being either a very common or a very rare mode of behavioural evolution. The genetic architecture of behaviour—polygenic and rich in epistatic interactions—creates the potential for a vast array of possible presuppressive interactions. Furthermore, with behavioural traits, epistasis can occur not only within genomes, but also between them (e.g., Heath 2010; Linksvayer 2007). Between‐individual epistasis is a common property of social systems (Sinervo et al. 2008; Wolf 2000) that greatly increases the total number of interactions (e.g., between parents and offspring, queens and workers, etc.) that could presuppress deleterious mutations.

On the other hand, a sceptic of CNE's role in behavioural evolution could argue that while the set of possible interactions is much greater for traits impacted by many genes (like behaviour) than those impacted by a few (like a single protein complex), the subset of these interactions that could meet the requirements for CNE is much smaller. In CNE, presuppressors must render otherwise deleterious changes neutral: while it is easy to imagine this occurring when, say, a protein binds to and stabilises a small stretch of RNA, this is harder to do for whole organism behaviours. Whole organism behaviours require more energy and resources to perform—perhaps there are fewer truly 'neutral' behavioural variants than subcellular variants? If so, the frequency of CNE would be much lower at the level of behaviour than at subcellular levels. Similarly, it could be argued that single conditionally neutral behaviours (such as darting in stingless bees) are less likely to be underpinned by a single mutation than, say, changes to protein structure in molecular CNE. However, variation at even a single genetic locus can contribute significantly to behavioural variation, such that single mutations could lead to the presence or absence of a particular behaviour (Gloria‐Soria and Azevedo 2008; Massaro et al. 2013; Osborne et al. 1997; Yagound et al. 2020; Zuk et al. 2014). We also note that the CNE hypothesis makes no claim about the effect size of conditionally neutral mutations. While, for ease of explanation, we have considered the spread of single macromutations in our hypothetical case studies, CNE could equally proceed with the accumulation of many conditionally neutral mutations of small effect.

Another approach to evaluating the likelihood of behavioural CNE is to consider which behaviours might be particularly prone to CNE‐driven complexification. While all behaviours have the potential to alter the selective pressures a population experiences (Odling‐Smee et al. 1996), cooperative interactions are of particular interest due to their potential to render mutations conditionally neutral and promote further dependencies between individuals (such as parental care, discussed above). Behaviours that allow organisms to buffer physiological stress, such as behavioural thermoregulation, similarly can allow otherwise‐deleterious mutations to spread (Bogert 1949; Huey et al. 2003; Muñoz 2022). For example, theory predicts that the evolution of huddling behaviour leads to relaxed selection on individuals to limit thermal conductance (e.g., reducing individual fat/fur levels), such that individuals become dependent on one another for a function they could previously perform independently (Glancy et al. 2016). Relaxed selection could also be driven by strong motivational states, as illustrated by the POP rituals of stingless bees. Further afield, Deacon (1998, 2010, 2017) has argued that the complexity of human language should be seen not as evidence of strong positive selection but actually for strongly relaxed selection. Cultural systems can also undergo CNE‐driven complexification (Appendix S2).

The flexibility and plasticity inherent to behaviour not only likely have roles in adaptive evolution, but also mean that behavioural plasticity can ameliorate costs of otherwise‐deleterious genetic mutations (phenotypic accommodation; West‐Eberhard 2003, 2005). Phenotypic accommodation thus acts as a presuppressor. CNE could occur both in cases where the behavioural capacity for presuppression is itself an evolved adaptation (e.g., chaperone proteins at the molecular level; adaptive plasticity at the behavioural level) and cases where the capacity for presuppression is coincidental (e.g., coincidental binding between proteins at the molecular level; coincidental temporal co‐occurrence or nonadaptive plasticity at the behavioural level). The difficulties in predicting how common behavioural CNE may be are compounded by biologists' failure to agree on what behaviour even is (Levitis et al. 2009). One could argue that many CNE models already published do actually describe behaviour, albeit not that of whole organisms: the behaviour of large multiprotein complexes such as the spliceosome, RNA editing machinery and ribosomes.

### 3.5 Testing the Behavioural CNE Hypothesis

The behavioural CNE hypothesis makes testable predictions. For instance, increases in behavioural complexity via CNE should leave genomic signatures of relaxed, rather than positive, selection. Such associations between relaxed selection and increases in social complexity are increasingly being identified. Fletcher (2023) found that the convergent evolution of maternal care in treehoppers is persistently accompanied by pronounced relaxed selection. The degree of social complexity is associated with relaxed selection in solitary, primitively eusocial and advanced eusocial insects (Imrit et al. 2020; Kapheim et al. 2015).

If a trait evolves via CNE, differences in its complexity between lineages should correlate better with the strength of genetic drift than with ecological or social factors. When drift is strong, the conditionally neutral (presuppressed) mutations that increase complexity can fix quickly. Ord and Garcia‐Porta (2012) provide striking evidence for this pattern: neutral phylogenetic models consistently outperformed adaptive models in predicting the level of signal complexity in sets of closely related species (Table 1). This is consistent with the prediction of CNE that changes in the strength of genetic drift should determine the fixation rate of conditionally neutral mutations that increase complexity.

**TABLE 1:** Relative ranking of the null phylogenetic model against selective models for a range of signals as tested by Ord and Garcia‐Porta (2012).

Taxon	Signal variable	Number of models run	Ranking of the null phylogenetic model
Frogs	Call amplitude modulation	7	1
Frogs	Call duration	7	2
Birds (distantly related)	Syllable repertoire size	6	2
Birds (distantly related)	Song repertoire size	6	1
Birds (closely related)	Syllable repertoire	4	2
Birds (closely related)	Syllable duration	4	1
Birds (closely related)	Song duration	4	1
Lizards (distantly related)	Number of ornaments	5	3
Lizards (distantly related)	Colour dichromatism (exposed)	5	1
Lizards (distantly related)	Colour dichromatism (concealed)	5	2
Lizards (closely related)	Number of head bobs	7	3
Lizards (closely related)	Display duration	7	1
Ants	Number of different cuticular hydrocarbons	5	1

*Note:* for example, when comparing variation in cuticular hydrocarbon number across ant species, five models were run: two ‘social’ (colony size; the social mating system), two ‘ecological’ (rainfall; temperature), and a null phylogenetic (neutral) model. Only one neutral model was ever run. The lower the rank, the better the model (i.e., if a model ranked in position one, it performed best). If behavioural complexity is increasing via CNE, the null model should perform well.

Other studies have also found that changes in complexity correlate better with levels of genetic drift than ecological factors (Table 2). Moreover, there are many cases where drift‐driven divergence in complexity seems likely despite a phylogenetic comparison between a neutral and an adaptive hypothesis not being made (Deacon 2017; Freeberg et al. 2012; McCracken and Sheldon 1997; Price and Lanyon 2002; Wischmann et al. 2012).

**TABLE 2:** Compilation of cases from the literature where differences in signal complexity could not be explained by functional/adaptive hypotheses, and instead appeared to correlate with levels of genetic drift—as predicted by the behavioural CNE hypothesis.

Taxon	Signal and the basis of interpopulation differences in complexity	Functional hypotheses tested?	Evidence for drift‐driven evolutionary divergence?	Notes	Key reference
Lemuroidea (lemurs)	Facial colour pattern complexity (total number of different hair colours in all areas)	Group size ✘ Number of sympatric species ✘ Climatic factors**?**	✔	Climatic factors affected complexity in a few facial regions, but moderate to high values of Pagel's lambda were found in almost all models	Rakotonirina et al. (2017)
*Phylloscopus trochiloides* (greenish warbler) species complex	Calls that differ in ‘stroke’ number	Morphological constraint ✘ Sensory drive ✘	✔		Irwin et al. (2008)
*Campylorhynchus rufinucha* (Mesoamerican rufous‐naped wren)	Calls that differ in phrase number and other structural characteristics	Adaptation to climatic differences ✘	✔		Sosa‐López et al. (2013)
*Allobates femoralis* (brilliant‐thighed poison frog)	Calls that differ in note number	Character displacement ✘	✔		Amézquita et al. (2009)
*Hirundo rustica* (barn swallow)	Calls that differ in pulse number	Climatic adaptation ✘	✔		Wilkins et al. (2018)
Dendrobatidae (poison‐dart frogs)	Calls that differ in pulse number	Temperature ✘ Body size ✘	✔		Erdtmann and Amézquita (2009)

*Note:* Several studies find a joint role for adaptation and drift (e.g., Ord and Martins 2006); while CNE may work alongside natural selection in increasing complexity, here we only include cases where neutral hypotheses are supported and adaptive hypotheses are not supported. A ✘ denotes a hypothesis that was not supported, a ✔ denotes a hypothesis that was supported, a **?** denotes a hypothesis that was partly supported.

The evidence collated in Tables 1 and 2 suggests that drift‐driven changes in behavioural complexity are taxonomically widespread. The CNE hypothesis provides a possible explanation for these patterns, though it need not be the only explanation and remains to be tested. Reconstructing ancestral character states offers another avenue by which CNE hypotheses can be tested, as illustrated by Finnigan et al.'s (2012) demonstration that increases in vacuolar H^+^‐ATPase complexity did not increase its function. However, the ideal evidence for behavioural CNE would be either observing the process directly (Després et al. 2024) or identifying genomic signatures unambiguously associated with CNE.

## 4 Conclusion

Natural selection provides a compelling explanation for the origin and increase of functional complexity, but molecular biologists are increasingly asking whether all complexity is functional. Constructive neutral evolution explains how nonfunctional complexity could arise and increase over time. Evolutionary biology, however, currently contains an unrecognised and unjustified disparity: the belief that the complexity of subcellular traits can be increased by selection and CNE, whereas the complexity of whole organism traits can only be increased by selection. Determining the relative impact of neutral and selective forces on different areas of the phenotype remains an important question in evolutionary biology.

We have explained how CNE could operate at the level of behaviour and argued that behaviour is particularly susceptible to CNE due to the number and types of interactions between components in behavioural systems. Moreover, behavioural traits have a capacity to act as presuppressors, by shielding organisms from some selection pressures.

Evidence for behavioural CNE is currently limited. In some cases, evolutionary increases in behavioural complexity seem better explained by neutral drift‐based models than by selective models. Verbal arguments also suggest that many behavioural traits could function as presuppressors, such as those associated with parental care or behavioural thermoregulation. These systems are good candidates for behavioural ecologists to test for evidence of CNE.

The understanding that CNE can produce ‘non‐functional complexity’ has generated novel hypotheses for the origin of complex cellular phenotypes. We believe that the study of the evolution of behaviour will be similarly enriched by acknowledging the possibility that, due to CNE, not all behavioural complexity is necessarily functional.

## Author Contributions

**Andrew M. Catherall‐Ostler:** conceptualization (lead), visualization (equal), writing – original draft (equal), writing – review and editing (equal). **Tanmay Dixit:** conceptualization (supporting), funding acquisition (lead), visualization (equal), writing – original draft (equal), writing – review and editing (equal).

## Conflicts of Interest

The authors declare no conflicts of interest.

## Supporting information


Appendix S1

## Data Availability

The authors have nothing to report.
