# Supplementary material for: The Constructive Neutral Evolution of Behaviour
Source: Ecol Evol. 2025 Jul 10;15(7):e71736. doi: 10.1002/ece3.71736 (PMC12245481; doi:10.1002/ece3.71736)
Supplement: Supplementary file 1 — Appendix S1 [file ECE3-15-e71736-s001.docx]

**APPENDIX S1**

**The evolution of hypnotic akinesis via CNE and other explanations for POP evolution**

In the main text of the manuscript, we explain how stingless bees (Meliponini) are characterised by a remarkably complex provisioning and oviposition process (POP). There is a huge diversity of POP rituals among meliponines, with many different styles of these ‘symbolic conversations’ (Zucchi et al., 1999) seeming to accomplish essentially the same task. We argue that the behavioural complexity that characterises these rituals could be explained by CNE and discuss how darting may have arisen via a process in which the queen’s response rules could act as a presuppressor.

Another behaviour for which CNE seems a particularly promising candidate explanation is ‘hypnotic akinesis’: when in front of the queen, workers of many stingless bee species will lower their heads before, as if hypnotised, slowly turning and then freezing still for a period of time (akinesis; Drumond et al., 2000). Little has been written about this behaviour, but it provides a good example of a behaviour in which non-functional hypotheses for its origin seem reasonable to consider. Below we describe how hypnotic akinesis (hereafter HA) could have arisen via CNE due to the worker’s behavioural rules acting as a presuppressor for mutations that affect queen behaviour.

Ordinarily, a mutation that made workers ‘akinetic’ would surely be selected against: it is hard to envisage why a worker would benefit from stopping the task it was doing and standing still, or how the queen could benefit from inducing this behaviour in her workers. But if HA only occurred immediately before or after the worker antennates the queen in the POP, then it would probably be cost-free. This is because the worker has just done the discrete piece of ‘work’ required of it in this instance: the queen has been informed and will shortly trigger the next phase of the POP. A short period of hypnotic akinesis at this point in the process may not be functional, but it is probably harmless. The temporal co-occurrence with the worker’s useful antennation shields the hypnotic akinesis mutation from negative selection. A mutation resulting in hypnotic akinesis would be unlikely to spread without such a ‘shielding’ association – if the worker froze during the course of her normal brood care or defensive activities, for example, this would surely be costly and selected against. Whilst reversional mutation is possible, further mutations could then easily drift to fixation – e.g. the queen tapping workers during akinesis in *Melipona*, the variable presence of worker metasomal bending or raising in *Paratrigona*, or the mounting of the akinetic worker in *Trigona spinipes*.

An alternative hypothesis for POP rituals is that these behaviours are ritualised aspects of the queen-worker conflict (Zucchi, 1993) – their function is to help queens and workers promote their own genetic interests. There are a number of problems with this hypothesis however: the frequency of darting and lunging during the POP does not correlate with levels of queen-worker conflict (Tóth et al., 2003), and these allegedly ‘aggressive’ behaviours never actually stop the provisioning and oviposition process from being performed. The outcome is always the same, and neither workers nor queens are reported to be injured during the interactions. Zucchi (1993), a strong supporter of the conflict interpretation for POP behaviours, describes *Plebeia* queens as begging for food aggressively, with the worker being seized and held down, but acknowledges that despite these frequent ‘aggressive’ behaviours, actual food delivery is rare. The hypothesis that these behaviours have no useful function, and instead have evolved because their time of expression made them essentially cost-free, helps explain these observations as well as offering a non-adaptive hypothesis for high levels of inter/intraspecific POP diversity.

Sakagami (1982)’s hypothesis for the origin of POP behaviours can also be phrased in terms of CNE. Sakagami argued that the ancestral state was a colony in which the oviposition rate was low. Increases in queen oviposition rate would have led to conflict between queens and workers. Workers provisioning and constructing brood cells would increasingly find themselves in close proximity to a waiting queen. Queens are behaviourally dominant, and as a queen approaches the worker this stimulates conflicting motivational drives to both attack and flee from the queen.

The drive to flee (F) is generally higher than the drive to attack (A) – and this relation (F > A) had a pre-suppressive effect on the interaction between waiting queens and provisioning workers. The conflict between F and A leads to the ritualised aggressive behaviours exhibited by workers (e.g. dart-and-retreat), but because F > A, the queens and workers do not enter actual costly combat. If A > F, then the increase in oviposition rate could not have occurred. The arrival of a queen led to provisioning workers rapidly exiting the cell (due to strong F), but also stimulated a weak A which leads to the pointless-but-harmless behaviours seen in the POP ritual. “The primordium of complicated oviposition behaviour was established by the linkage” of the arrival of a waiting queen and the relation F > A (Sakagami, 1982). The additional behaviours that characterise the POP do not increase the functionality of the colony – they are the nonadaptive byproducts of a system in which their expression is not costly, thanks to the pre-suppression provided by the relation F > A and the strong drive of the queen to oviposit. In other words, a mutation that led to darting during the POP would be (conditionally) neutral if F > A and the queen was motivated to oviposit, but not if A > F and/or the queen was not motivated to oviposit.

**APPENDIX S2**

**Cultural CNE**

If genetic evolution can occur via CNE, could culture also undergo CNE-driven complexification, leading to cultural practices becoming more complex without becoming more functional? A comparison of ‘genetic’ CNE with cultural CNE (Supplementary Table 1) reveals the primary difference is in the nature of the ratchet. Whereas in genetic CNE the ratchet arises from the distribution of future mutational effects, in cultural CNE the ratchet could arise from a low probability of cultural reversion, for example the tendency of species such as humans to prefer makeshift or improvised ‘workarounds’ to deal with challenges to an existing system rather than designing an entirely new system. Such ‘workarounds’ may lead to a small increase in complexity, but avoid expending the time and energy that must be invested to redesign the whole system. The latter will normally only be preferred where workarounds do not exist or are inadequate in some way; this leads us to expect that ‘workarounds’ that do the job just as well, albeit less simply, to accrue within a system. The same occurs in biological systems –evolution is a tinkerer, not an engineer (Jacob, 1977). Just as more and more RNA editing sites accumulate in a protist, so do more and more exceptions and peculiarities in human cultural systems.

Supplementary Table 1 – a comparison between biological and cultural CNE.

| **Stage** | **Genetic CNE** | **Cultural CNE** |
| --- | --- | --- |
| Starting point | All complexity in the system is functional | All complexity in the system is functional |
| Step 1 | A mutation occurs which adds complexity to the system and would normally be selected against… | A change occurs which would normally require the removal/revision of one of the system’s components… |
| Step 2 | …but is presuppressed by a fortuitous interaction with a pre-existing component | …but it is presuppressed by a fortuitous interaction with a pre-existing component |
| Step 3 | Nothing now stops the mutation from reaching fixation.   The system becomes more complex without any increase in function, as the system contains a new dependency. | This provides an alternative option to removing/revising a system’s component.   The system has become more complex without any increase in function, as the system contains a new dependency |
| Step 4 | Further increases in complexity can occur if the particularities of the system make further complexification more likely than mutational reversion | Further increases in complexity can occur if the particularities of the system make further complexification more likely than wholesale reform. |

A famous example of pointless complexity can be found in the House of Commons, the lower chamber of the Parliament of the United Kingdom. Members of the House of Commons (hereafter MPs), once elected, cannot resign. Despite being unable to legally resign, several MPs do actually leave the Commons voluntarily each Parliament (with a replacement being elected), thanks to a convoluted piece of legal fiction in which the MP is forced to ask the monarch to appoint them to an ‘office of profit under the crown’, and become either the ‘Crown Steward and Bailiff for the Chiltern Hundreds’ or the ‘Crown Steward and Bailiff of the Manor of Northstead’.

The unnecessary degree of complexity involved in what could (and should) really be a very simple process has long been noted:

“…the long and short of it is, that in its present development the Stewardship of the Chiltern Hundreds is as bewildering an anomaly as you can find on a summer’s day. Any account of it reads like an unwritten chapter of Rabelais ‘How Pantagruel arrived at the Island of Paradox’. In the eye of the law an office, it [the Stewardship] has no official duties, functions, or characteristics of any sort whatsoever; a post of profit, no vestige of emoulments attatches to it; granted by the Sovereign, Her Majesty has absolutely no voice in making the appointment. Formally nominated as the Crown’s agent over an estate where the Crown has no rights, the Steward has to account for moneys which he cannot receive, and to hold Courts which no suitor ever attends. In return he enjoys ‘wages, fees and allowances’ no more substantial than the Emperor’s new clothes in Hans Andersen’s story, and ‘privileges and pre-eminences’ are conferred upon him which exist nowhere outside of Nephelo-coccygia [cloud cuckoo land]…” - (Gifford et al., 1894)

We are not concerned with every constitutional oddity here, but it is interesting to treat this a test case in which to look for parallels between the evolution of unnecessary cultural complexity and the evolution of unnecessary biological complexity.

Originally, the prohibition on resignation made sense. Up until the eighteenth century, many MPs would have been all too willing to resign: parliamentary membership was often a burdensome obligation accepted reluctantly (Hicks, 2019). Being a parliamentarian meant long, unpaid journeys to Westminster via a poorly kept and dangerous road network. Hence the House resolved that “a man, after he is duly chosen, cannot relinquish”.

After the English Civil War, the House of Commons’ political power was greater than ever before, and this incentivised monarchs to try and get MPs elected who would be favourable to their agenda. The House of Commons was supposed to hold the Crown to account, but it began to contain many MPs whose loyalty to the Crown was bought through royal bribes of both money and offices (Gifford et al., 1894; Hicks, 2019).

Preventing the Commons from being filled with those also employed by the Crown was difficult. The Commons could not simply expel any person who already held an office under the Crown, as this would include all Government Ministers – the very people who the Commons was supposed to scrutinise. Instead, in 1707 the Commons solved this conundrum with the Place Act: any person newly appointed to an office under the Crown whilst an MP would automatically lose their seat in parliament. The Place Act was ingenious: any monarch trying to tempt a pliant MP with a bribe would fail, as the moment the MP received money from the monarch, they could no longer be an MP. Sincere appointments – e.g. a new Government Minister – could still occur, but would still require the MP to vacate their seat and re-run for election (Gifford et al., 1894; Hicks, 2019).

Over time it became clear that the Act had also, albeit unintentionally, created a new opportunity for MPs who *wanted* to vacate their seats, for whatever reason. The Place Act had within it an *unanticipated capacity* to assist them in doing so, but it would take some years before this would be realised. In 1750, John Pitt MP wished to resign his seat and asked the King to appoint him as the Steward of the Chiltern Hundreds. Previously, the Steward had to administer certain royal estates, but by the 1700s the office no longer had any actual duties or renumeration, existing only on paper. The essentially fictious appointment was made, and as it was technically a Crown office, John Pitt lost his seat just as he desired (Gifford et al., 1894; Hicks, 2019). Pitt had managed to do the impossible, resigning from an unresignable position – and Parliamentary procedure had gained a curious complexity.

After this first recognition of the Place Act’s unanticipated excess capacities, it was likely that the process was only going to complexity further. A new, grotesquely complex system quickly evolved: MPs wishing to resign could ask not only to ‘take the Chiltern Hundreds’, but could instead ask to be appointed the essentially fictious Crown Stewardships of either the Manor of Old Shoreham (Sussex), or the Manors of Poynings (Sussex), Hempholme (Yorkshire), East Hendred (Berkshire) etc; Irish MPs could be appointed to the Escheatorship of Munster or the Escheatorship of Ulster. Conventions even evolved to regulate the order of appointment to these imaginary offices; nowadays appointments are made to the Stewardships of the Chiltern Hundreds and the Manor of Northstead alternately to allow for the case where two MPs wish to resign at the same time (Gifford et al., 1894; Hicks, 2019).

No-one benefits from this complexity, and this system is no more functional in allowing MPs to leave the House of Commons than a system in which they simply write a letter of resignation. The origin of the complexity can be understood by a recognition that cultural systems can also undergo constructive neutral evolution.

**Starting point**: The ban on resignations and the later Place Act of 1707 were both functional piece of legislation passed to solve a real problem. The first ensured there would be a full House of Commons, the second that the monarch would find it difficult to buy the votes of parliamentarians. The two rules had a ‘neutral interaction’ – i.e. the Place Act did *potentially* allow for the resignation ban to be circumvented, but this was not *actually* used. It is analogous to a neutral biological interaction as the link between the two pieces of legislation was not providing a useful function.

**Step 1:** A change began to occur that threatened the survival of the current system, making the removal of one of its components likely. In this case, the change was the utility in the ban on MPs resigning: whilst originally useful it was now an encumbrance (as many new candidates would be happy to take the place of an MP who wished to resign).

**Step 2:** Normally, this reversal in the functional value of a rule or piece of legislation would lead to its repeal. But in this case, another unrelated component in the system fortuitously watered down the negative consequences of the threatened component. In other words, the Place Act’s wording had the unintended consequences that a Crown ‘appointment’ to an essentially fictitious office that existed only on paper would cause an MP to lose their seat. This allowed the ‘MPs cannot resign’ rule to survive.

**Step 3**: The system had thus become more complex: the ‘MPs cannot resign’ rule was now dependent on the previously unrelated Place Act for survival, as well as the on-paper existence of certain Crown sinecures. Overall, however, the system had become no more functional than one in which MPs simply resign.

**Step 4:** Of course, it would have been entirely possible for MPs to simply repeal the ‘MPs cannot resign’ rule, should they have wished. But as they chose not to, the system could rapidly evolve further pointless complexity, as there were far more ways for it to become more complex than to become simpler. The Place Act did not specify the Stewardship of the Chiltern Hundreds in particular, and so other offices were added: the Stewardship of the Manor of Northstead, the Escheatorship of Munster, etc. The Place Act’s unexpected consequences allowed many more on-paper-only sinecures to be recruited into the system. More and more arcane appointments became available, shielded from scrutiny due to their inconsequentiality.

In other words, the system of resignation from the British House of Commons evolved due to fortuitous pre-suppression preventing the removal of superfluous components and instead permitting these and further complexities to accumulate, without any increase in overall function – a process strikingly similar to CNE. Similar accounts can be given for many similar ‘legal fictions’. Much like the complexities of the eukaryotic clock or RNA editing processes, the evolution of the resignation procedures from the House of Commons show how some components may play a role in a system because the system got stuck with them, not because they had a useful function. They have made themselves necessary.

**References**

Drumond, P. M., Zucchi, R., & Oldroyd, B. P. (2000). Description of the cell provisioning and oviposition process of seven species of Plebeia Schwarz (Apidae, Meliponini), with notes on their phylogeny and taxonomy: *Insectes Sociaux*, *47*(2), 99–112. https://doi.org/10.1007/PL00001703

Gifford, W., Coleridge, S. J. T., Lockhart, J. G., Elwin, W., Macpherson, W., Smith, W., IV, S. J. M., & Ernle), R. E. P. (Baron. (1894). *The Quarterly Review*. John Murray.
Google-Books-ID: D7M_AQAAMAAJ

Hicks, E. (2019). *Resignation from the House of Commons* (Briefing Paper (House of Commons Library) 06395; House of Commons Library).

Jacob, F. (1977). Evolution and Tinkering. *Science*, *196*(4295), 1161–1166. https://doi.org/10.1126/science.860134

Sakagami, S. F. (1982). Stingless bees. *Social Insects*, *3*, 361–423. https://doi.org/10.1016/b978-0-12-342203-3.50011-4

Tóth, E., Strassmann, J. E., Imperatriz-Fonseca, V. L., & Queller, D. C. (2003). Queens, not workers, produce the males in the stingless bee Schwarziana quadripunctata quadripunctata. *Animal Behaviour*, *66*(2), 359–368. https://doi.org/10.1006/anbe.2003.2218

Zucchi, R. (1993). Ritualized dominance, evolution of queenworker interactions and related aspects in stingless bees (Hymenoptera, Apidae). *Evolution of the Insect Societies, Comparative Sociology of Bees, Wasps and Ants*, 207–249.

Zucchi, R., Silva-Matos, E. V., Nogueira-Ferreira, F., & Azevedo, G. (1999). On the cell Provisioning and Oviposition Process (POP) of the stingless bees—Nomenclature reappraisal and evolutionary considerations (Hymenoptera, Apidae, Meliponinae). *Sociobiology*, *34*, 65–86.
